# Ultrasonography for the Diagnosis of Intussusception in Children: An Experience From Pakistan

**DOI:** 10.7759/cureus.9656

**Published:** 2020-08-11

**Authors:** Arthina Dadlani, Sajan Lal, Bhesham Shahani, Muhammad Ali

**Affiliations:** 1 Radiology, Dr. Ziauddin Hospital, Karachi, PAK

**Keywords:** intussusception, ultrasonography, bowel obstruction

## Abstract

Introduction: Intussusception can lead to small bowel obstruction in children, hence the early diagnosis of this condition is very important. The purpose of this study is to evaluate the accuracy of sonography in the diagnostic work-up of children with suspected intussusceptions in the emergency setting, keeping surgical findings as the gold standard.

Methods and design: Two hundred patients with classical presentation of intussusceptions, who were diagnosed either by barium enema or CT scan, were included in this study. Patients with irreducible intussusceptions on color Doppler were followed after surgery.

Results: The average age of the patients was 6.7 ± 2.8 years, and the study population consisted of 115 (57.5%) boys and 85 (42.5%) girls. One hundred forty-three patients were confirmed to have intussusception on ultrasonography, of whom 117 (81.8%) were confirmed to have intussusception after surgery while 26 (18%) were not diagnosed with intussusception during surgery. Despite the clinical presentation of intussusceptions, ultrasonography was not diagnostic in 57 patients, of whom 28 were confirmed to have intussusception after surgery and 29 were not found to have the disease.

Conclusion: Use of ultrasonography in cases with intussusception has proven to be a reliable and accurate method for diagnosing intussusception and provides an advantage over unnecessary radiological or surgical procedures being performed.

## Introduction

Intussusception is a common cause of children requiring admission to the emergency department and can lead to bowel obstruction. Intussusception occurs when a segment of the bowel invaginates into an immediately adjacent segment, often likened to a telescope, resulting in obstruction of the bowel, ischemia, and necrosis [1,2]. Five types of intussusceptions are described: ileo-colic, ileo-ileo-colic, jejuno-jejunal, jejuno-ileal, and colo-colic. Ileo-colic intussusception is the most common type, accounting for over 80% of cases in children [3].

Most of the symptoms are due to obstruction of the bowel and consist of a classic triad of abdominal colic, bile-stained vomiting, and red jelly stools. This triad of intermittent symptoms has a positive predictive value of 93%. Adding the symptom of rectal bleeding to this triad raises the positive predictive value to 100% [4].

Intussusceptions are one of the most common causes of bowel obstruction in infancy. It can lead to intestinal necrosis, bowel resection, and even death if not recognized and treated appropriately. Intussusception seems to be idiopathic in 90% of cases and is associated with pathologic lead points such as Meckel’s diverticulum, solid bowel lesion, and intestinal lymphoma [5-6]. It can occur postoperatively and after blunt abdominal trauma [7].

Intussusception in infants occurs at 0.3 to 2.7 cases per 1,000 live births in Europe, North America, and Australia [8-9]. However, the incidence rate is higher in some developing countries, which leads to a higher rate of complications in those areas [10].

A diagnostic enema may be the most cost-effective method of excluding or confirming the diagnosis, particularly when air enema is used. Most children presenting with classic features of intussusceptions should undergo air enema directly to avoid duplication of investigation.

CT is a reliable method for diagnosing intussusception in adults. Abdominal radiographs should be reserved for children with clinical evidence of peritonitis and possible perforation, an atypical clinical presentation, or equivocal ultrasonography findings.

Abdominal ultrasonography is an excellent screening test for children with non-classical clinical features. Ultrasonography screening has been suggested to reduce cost, radiation exposure, and anxiety/discomfort of patients and parents. Published literature suggests high accuracy, approaching 100% in experienced hands, with sensitivity of 98% to 100% and specificity of 88% to 100% [11]. Additionally, ultrasonography helps in identifying alternative diagnoses as well as the evaluation of reducibility of an intussusception, the presence of a lead point mass, and intussusception limited to the small bowel [12,13].

The advantages of ultrasonography are that it allows noninvasive, rapid, and confident diagnosis with lack of ionizing radiation as compared to barium enema. It can be done bedside, even with less experienced readers.

Due to a scarcity of literature with relevant data, our aim is to present the available data at our center. The purpose of this study is to evaluate the accuracy of ultrasonography in the diagnostic work-up of children suspected of intussusception in the emergency setting, keeping surgical findings as the gold standard.

## Materials and methods

With approval from the Clinical Research Committee and Ethics Review Committee of Ziauddin University, we performed a retrospective review of the electronic medical records of 200 patients fulfilling the inclusion criteria from 31/08/2017 to 01/03/2018. Patients were included from inpatient, outpatient, and emergency department of Dr. Ziauddin Hospital. Inclusion criteria considered were: patients with suspicion of intussusception presenting with a clinical triad of colicky abdominal pain, vomiting, and red jelly stools, and children below 12 years of age of either sex.

Informed consent was taken from the parents over the phone to include the patient's data, and a brief medical history regarding duration and symptoms of intussusceptions was sought. All information gathered during this study was kept confidential.

All ultrasonography images were reviewed from the saved data. Ultrasonography was performed using probes of 3.5 MHz and 8.0 MHz on a Toshiba Aplio machine (Toshiba, Tokyo, Japan). Serial longitudinal and transverse images were taken and assessed by a senior attending radiologist with three years of experience in radiology. All patients diagnosed as having signs of irreducible intussusceptions, such as free fluid or absent blood flow on color Doppler, were followed after surgery. Those cases in which barium enema failed to reduce the intussusception, making surgery the ultimate treatment, were also included. Patients' findings (positive or negative) were collected and documented on a proforma attached with annexure.

Data analysis procedure

Patients' data were collected and analyzed through SPSS for Windows, Version 16.0 (SPSS Inc., Chicago, USA). Patients' ages were presented by mean±SD. After analyzing the data, sensitivity, and specificity, negative and positive predictive values of ultrasonography for detecting intussusceptions were calculated by taking surgical findings as the gold standard. Kappa analysis was also performed to measure the degree of agreement between surgical and ultrasonography findings, and a p-value less than 0.05 was considered significant.

## Results

Two hundred patients with intussusception diagnosed on ultrasonography confirmed either by barium enema or CT scan were included in this study. The average age of the patients was 6.7 ± 2.3 years (Table 1). There were 115 (57.5%) boys and 85 (42.5%) girls.

**Table 1:** Descriptive statistics of age

Demographics	
Mean age (Years)	6.7 ± 2.8
Male	115
Female	85
Ultrasonography positive	143
Ultrasonography negative	57

The rate of intussusception in children was 72.5% (145/200) confirmed after surgery while ultrasonography reported 71.5% (143/200) of patients with intussusception. Similarly, true-positive, true-negative, false-positive, and false-negative findings are reported in Table 2.

**Table 2:** Ultrasonography and surgical findings in the diagnostic work-up of children with suspected intussusception

Ultrasonography	Surgical Findings		Total
	Positive	Negative	
Positive	117 (TP)	26 (FP)	143 (71.5%)
Negative	28 (FN)	29 (TN)	57 (28.5%)
Total	145 (72.5%)	55 (27.5%)	200

Sensitivity, specificity, positive predictive value, negative predictive value, and accuracy of ultrasonography in detection of intussusception in children were 80.7% (95%CI: 73.5 to 86.3), 52.7% (95%CI: 39.79 to 65.31), 81.8% (95% CI: 74.7 to 87.3), 50.9% (95% CI: 38.3 to 63.4), and 73% (95%CI: 66.46 to 78.68), respectively, as shown in Table 3. Agreement was also observed that kappa statistics (k=0.33; p=0.005) showed the weak agreement between ultrasonography and surgical findings (Table 3).

**Table 3:** Diagnostic accuracy of sonography in the diagnostic work-up of children with suspected intussusception in the emergency setting, keeping surgical findings as the gold standard

Parameter	Estimate	95% CI
Sensitivity	80.7%	73.5 to 86.3
Specificity	52.7%	39.79 to 65.31
Positive predictive value	81.8%	74.7 to 87.3
Negative predictive value	50.9%	38.3 to 63.4
Diagnostic accuracy	73%	66.46 to 78.68
Cohen's kappa (unweighted)	0.33	0.19 to 0.47

**Figure 1:**
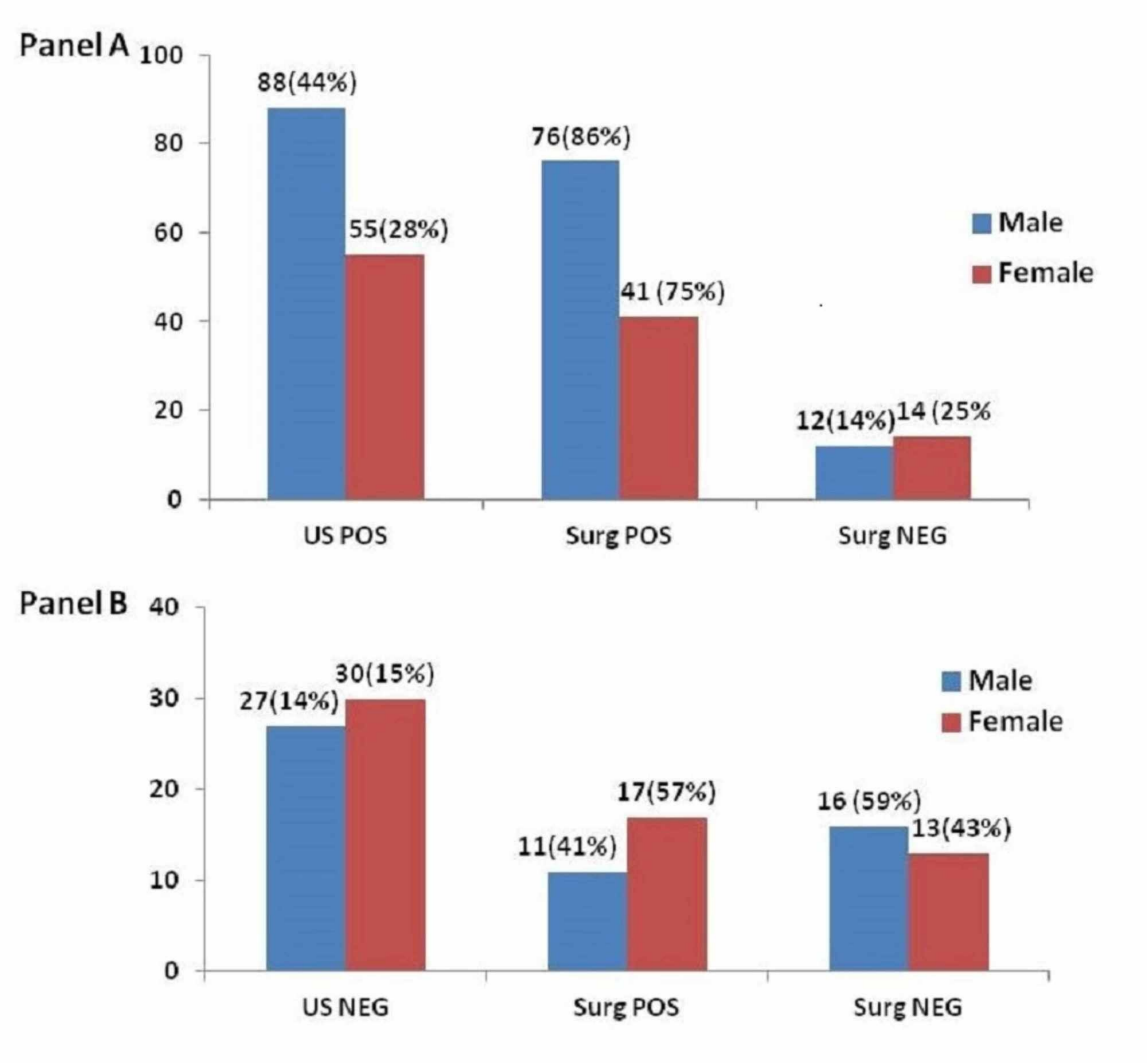
Distribution of intussusception in males and females (A) surgical confirmed (positive) and unconfirmed (negative) after intussusception diagnosed with US (US positive), (B) surgical confirmed (positive) and unconfirmed (negative) after ultrasonography was negative for intussusception (US negative) US pos: Ultrasound positive; US neg: Ultrasound negative; Surg pos: Surgery positive; Surg neg: Surgery negative

Diagnosis of intussusception was made with ultrasonography in 143 patients (71.5%), of whom 117 (81.8%) were confirmed to have intussusception after surgery while 26 (18%) were not diagnosed during surgery. Despite the clinical presentation of intussusception, ultrasonography was not diagnostic in 57 patients, of whom 28 were confirmed to have intussusception after surgery and 29 were not found to have the disease. Furthermore, data was analyzed separately in males and females. As shown in Figure 1, the ratio of males (88, 44%) diagnosed with ultrasonography was higher than females (55, 28%). 

## Discussion

The traditional investigations with small bowel enteroclysis and small bowel follow-through do not provide much information and unfortunately involve radiation exposure of the patient. Although it is an organ that is spared from frequent disease, more precise and patient-friendly methods are needed [14]. Recent advances in new imaging techniques, including CT, MRI, wireless capsule endoscopy, and double-balloon endoscope, have proven to be useful in diagnosing intussusception.

Ultrasonography is accurate and more affordable compared to other diagnostic modalities for intussusception; however, due to its dependency on operative experience, its reliability in diagnosing intussusception has been debated among radiologists. [15]. Ultrasonography is the primary imaging modality for initial diagnosis in developing countries. Ultrasonography also plays a role in the identification of alternative diagnoses, as well as the evaluation of reducibility of an intussusception, the presence of a lead point mass, and intussusception limited to the small bowel. Studies have shown that ultrasonography can lead to accurate (100%) diagnosis with experienced operators [13-16]. The advantages of ultrasonography are that it allows non-invasive, rapid, and confident diagnosis with lack of ionizing radiation as compared to CT scan and barium enema. It can be done bedside even with less experienced readers. It also helps to determine whether the involved bowel should be reduced non-operatively or surgically resected. Inability to detect blood flow in the intussusception predicts the need for surgery.

In this study, the average age of the patients was 6.69±2.27 years. There were 115 (57.5%) boys and 85 (42.5%) girls. In the Gul et al. study [17], 56.8% were boys, and 43.2% were girls. The boy to girl ratio was 1.3:1, and the mean age was 4.3±2.3 years.

In this study, sensitivity, specificity, positive predictive value, negative predictive value, and accuracy of ultrasonography in the detection of intussusception in children were 80.7% (95% CI: 73.5 to 86.3), 52.7% (95% CI: 39.8 to 65.3), 81.8% (95% CI: 74.7 to 87.3), 50.88% (95% CI: 38.3 to 63.4), and 73% (95% CI: 66.5 to 78.7), respectively. In our study, sensitivity in diagnosing intussusception was similar to other studies; however, specificity was low compared to available literature, likely due to operator experience, equipment availability, and urgent situations in which ultrasonography was performed.

In Gul et al., the overall sensitivity of ultrasonography in the detection of intussusception was 83.9%, specificity was 95.7%, and accuracy was 93.7% [17]. A large-scale prospective study reported that ultrasonography is 97.5% sensitive and 99% specific for the diagnosis of acute intussusception in children in developing countries, despite the use of equipment that was older than what is generally available in developed countries.

Ultrasonography is also useful in observing transient intussusception or spontaneous reduction obviating the need for enema reduction. Additionally, presence of other conditions including small bowel volvulus, necrotizing enterocolitis, and urinary tract disease can be detected with the help of ultrasonography at the same time [18-20].

Early diagnosis of intussusception with ultrasonography as an initial screening test will decrease the unnecessary radiation exposure with contrast-based enemas if ultrasonography is negative for intussusception. This may result in greater cost as a positive diagnosis on ultrasonography will subsequently require contrast enema as well. 

## Conclusions

In our study, ultrasonography was shown to be sensitive and accurate in diagnosing intussusception. It is a safe and valuable clinical tool in the diagnosis of acute intussusception in children due to its absence of radiation, non-invasiveness, and rapid nature. Additionally, our data can set the stage for physicians to confidently use ultrasonography in patients clinically suspicious for intussusception as the initial imaging modality over unnecessary radiological or surgical procedures, given its cost-effectiveness as well.
